# The Book World of Medicine and Science

**Published:** 1907-02-09

**Authors:** 


### The Book World of Medicine and Science.

Fibroid Tumour. Without Operation. By John Shaw, M.D. (Swan, Sonnenschein and Co., pp. 80. Illustrations 2.)

The occasion of the publication of this little book is duly set forth by Dr. Shaw in his preface. It appears that, in 1904, Dr. Shaw submitted to the editor of the *British Medical Journal* "a paper describing a non-operative treatment of certain diseases of women . . . such as it is the fashion to deal with by mutilative operations." The paper was subsequently returned with the intimation that the editor "saw no prospect of being able to publish his paper at an early date," and later "met with the same fate" at the hands of the editor of the *Medical Press and Circular.* The preface concludes: "As I have been unable to reach the ear of the medical profession (and thus the public) through the ordinary professional channels, it is my hope to do so through the medium of the present essay." Unfortunately the book is somewhat of a philippic against the recognised operative treatment, with an occasional word for the *British Medical Journal.* The mental attitude of the writer is indicated by the frequent occurrence of the expression, "mutilative operations," and by the following paragraph on operative statistics: "I gladly admit that there are a few operators whose published statistics are more favourable as to primary mortality than those which I am about to quote. But such success being, in my judgment, quite exceptional, it would be no wiser to accept it as a motive for action, or the basis of argument, than it would be for wild game to regard the well-being of a decoy duck as an adequate guarantee for the like conditions favouring the birds attracted by it." Having been "unable to reach the ear of the profession (and thus the public) through the ordinary professional channels," Dr. Shaw frankly addresses himself to the lay reader in the first sentence of the first chapter thus: "For the convenience of the non-medical reader . . . it will be desirable to explain certain technical terms." These include such words as *abdomen* and *womb*, and the chapter closes with a reference to the glossary at the end of the book. On turning to the glossary we find the following playful excursion into philology: "Womb. Originally the belly: the place where the young are conceived and kept till birth. *The word 'woman' is perhaps connected with 'womb.'*" Perhaps! but woman is Anglo-Saxon *wifman* from *wif*, meaning wife; while womb is the same word as old German *wambe*, from a root, common to all Indogermanic languages. For what class of reader has the book been written?

